# Marine-Derived Leads as Anticancer Candidates by Disrupting Hypoxic Signaling through Hypoxia-Inducible Factors Inhibition

**DOI:** 10.3390/md22040143

**Published:** 2024-03-23

**Authors:** Maria Rita Garcia, Paula B. Andrade, Florence Lefranc, Nelson G. M. Gomes

**Affiliations:** 1 REQUIMTE/LAQV, Laboratório de Farmacognosia, Departamento de Química, Faculdade de Farmácia, Universidade do Porto, 4050-313 Porto, Portugal; ritagarcia7@hotmail.com (M.R.G.); pandrade@ff.up.pt (P.B.A.); 2 1H-TOXRUN-Toxicology Research Unit, University Institute of Health Sciences, CESPU, CRL, 4585-116 Gandra, Portugal; 3 UCIBIO/REQUIMTE, Laboratory of Toxicology, Faculty of Pharmacy, University of Porto, 4050-313 Porto, Portugal; 4 Department of Neurosurgery, Hôpital Universitaire de Bruxelles (H.U.B), CUB Hôpital Erasme, Université Libre de Bruxelles (ULB), 1070 Brussels, Belgium; florence.lefranc@hubruxelles.be

**Keywords:** caulerpin, discorhabdin, dolastatin-15, echinomycin, epolactaene, fascaplysin, kalkitoxin, latrunculin, plitidepsin, psammaplin

## Abstract

The inadequate vascularization seen in fast-growing solid tumors gives rise to hypoxic areas, fostering specific changes in gene expression that bolster tumor cell survival and metastasis, ultimately leading to unfavorable clinical prognoses across different cancer types. Hypoxia-inducible factors (HIF-1 and HIF-2) emerge as druggable pivotal players orchestrating tumor metastasis and angiogenesis, thus positioning them as prime targets for cancer treatment. A range of HIF inhibitors, notably natural compounds originating from marine organisms, exhibit encouraging anticancer properties, underscoring their significance as promising therapeutic options. Bioprospection of the marine environment is now a well-settled approach to the discovery and development of anticancer agents that might have their medicinal chemistry developed into clinical candidates. However, despite the massive increase in the number of marine natural products classified as ‘anticancer leads,’ most of which correspond to general cytotoxic agents, and only a few have been characterized regarding their molecular targets and mechanisms of action. The current review presents a critical analysis of inhibitors of HIF-1 and HIF-2 and hypoxia-selective compounds that have been sourced from marine organisms and that might act as new chemotherapeutic candidates or serve as templates for the development of structurally similar derivatives with improved anticancer efficacy.

## 1. Introduction

### 1.1. Marine Natural Products in Cancer Therapy

Bioprospection of the marine environment has emerged as a new frontier in drug development due to the nearly unlimited potential of marine organisms as sources of lead structures that cover a wide range of pharmacological effects and biotechnological applications [1,2,3]. The vast repertoire of complex compounds with unconventional structural architectures, many possessing relevant and specific biological effects, turned the attention of organic chemists and pharmacologists to the depths of the oceans, leading to an exponential growth in the discovery of new marine natural products on the last 20 years [1,2,4,5,6].

Many of these compounds have been portrayed as ‘chemical weapons’ as they evolved to interact efficiently with biological targets, displaying an inherent degree of drug-likeness and frequently exerting relevant anticancer, antimicrobial, and immunosuppressive properties. For example, it is hypothesized that the decreased frequency of tumors in marine invertebrates may derive from an innate immune system [7]. Furthermore, there is also evidence that there is an inherent ability to overcome the sea dilution effect, which may partially explain the higher incidence of significant bioactivity compared with organisms from the terrestrial environment, with approximately half of the novel marine-derived natural products displaying biological activity [1,2,8,9].

It is clear that natural products have been the most successful source of anticancer agents ever [10,11,12]. Newman and Cragg have been updating the contribution of natural products to the development of drugs, with their latest review indicating that from all the chemotherapeutic drugs approved between 1946 and 2019, only 21% can be ascribed as truly synthetic, corroborating the major contribution of natural sources [13]. 

While most sources inspiring the development of anticancer agents have a terrestrial origin, marine-derived compounds are marking a milestone, providing new lead structures with unprecedented chemical diversity, striking anticancer properties and inspiring the design of several derivatives that keep feeding a constantly active marine pharmaceutical pipeline [1,13]. Cytarabine (Cytosar-U^®^) is traced back as the first marine-derived drug to receive market authorization from the Food and Drug Administration (FDA) in 1969. The development of cytarabine was inspired by the structural framework of two arabinose-containing nucleosides isolated from the sponge *Cryptotethya crypta* and completely transformed the approach to treating and handling hematological malignancies [14,15]. More than 50 years after cytarabine’s approval, thirteen additional marine-derived drugs received market approval, with 38 candidates currently in clinical development [16]. As reviewed by us in 2019, the clinical pipeline of marine-derived drugs, consisting of approved drugs and clinical candidates, has been particularly fruitful in cancer therapy, not only enabling the broadening of the scope of action in cancer treatment but also the discovery of new mechanisms of action and molecular targets [17].

Despite the apparent and exciting richness of new potential anticancer agents from marine sources [17,18,19,20,21], it should be taken into account that the early research on the chemistry of marine natural products was mainly directed to the identification of novel chemical structures rather than in their potential biological properties [22,23]. Early anticancer screening has been mainly focused on the mere evaluation of the cytotoxic properties against cancer cell lines, with no emphasis on the effective anticancer activity against multidrug-resistant (MDR) cell lines, selectivity, or elucidation of their mechanisms of action [1,2]. Consequently, only a disappointing fraction of compounds, determined as cytotoxic against human cancer cell lines, ultimately displayed in vivo activity and, subsequently, clinical relevance [24,25]. Indeed, the complexity of a tumor derives from the continuous crosstalk between the tumor cells and the microenvironment over time, adding the complication of temporal heterogeneity on top of spatial heterogeneity, and by no means a purely cytotoxic or proapoptotic agent is likely to translate into an anticancer drug [26]. Conceivably, distinct environmental landscapes within a given tumor select for mutations that engender survival and expansion, thereby creating tumor cell heterogeneity and a complex regional difference in selective pressures, including hypoxia, that actively shapes tumor development [26,27].

### 1.2. Hypoxic Signaling in Cancer Development

Due to insufficient vascularization, hypoxic regions form within rapidly growing solid tumor masses, specific alterations of gene expression in these hypoxic tumor cells helping to facilitate the survival and metastatic spread of solid tumors, being therefore associated with poor clinical outcomes in many types of human cancers [28,29,30]. Hypoxic cancer cells are, in fact, resistant to radiotherapy, chemotherapy, and targeted therapy [28,31,32]. 

**Figure 1:**
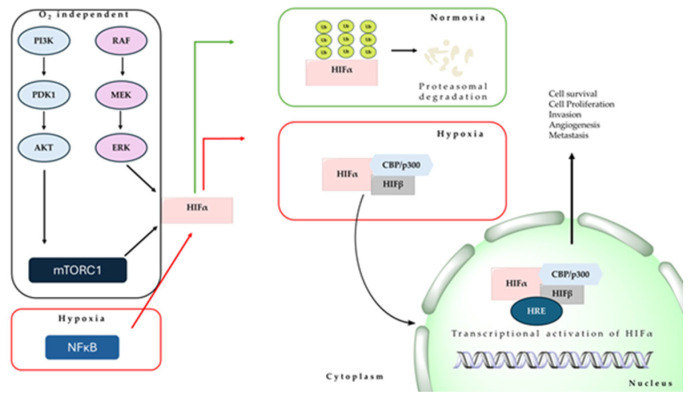
Pathways on hypoxia-inducible factor activation.

The hypoxic environment sparks cancer development by inducing an intricate cellular signaling network within cancer cells, encompassing the HIF, PI3K, MAPK, and NFĸB pathways (Figure 1). These pathways interact with one another, leading to the formation of both positive and negative feedback loops, ultimately amplifying or reducing the impact of hypoxic conditions [33,34]. Hypoxia triggers the activation of HIFs, these factors consisting of hypoxia-regulated α and oxygen-insensitive β subunits, playing a crucial role in regulating gene expression during hypoxia, both in normal and solid tumor tissues (Figure 1) [28,35]. The HIF family comprises three members: HIF-1α, HIF-2α, and HIF-3α, each serving distinct functions. Unlike HIF-1α and HIF-2α, HIF-3α exhibits variations in protein structure and gene expression regulation. During hypoxia, HIF-3α exerts a transcriptional regulatory function that negatively impacts gene expression by competing with HIF-1α and HIF-2α for binding to transcriptional elements in target genes [35,36]. Additionally, HIF-1α plays a significant role in regulating mitochondrial homeostasis, as one of the essential adaptations to sustained hypoxia involves suppressing mitochondrial respiration and inducing glycolysis [37,38]. On the other hand, the activation of HIF-2α enhances peroxisome turnover through pexophagy, leading to changes in lipid composition akin to peroxisomal disorders [39,40,41].

Activation of HIFs leads to a range of molecular effects, resulting in the transcription of numerous genes that play pivotal roles in processes such as angiogenesis, specification of cancer stem cells, cell motility, epithelial-mesenchymal transition, remodeling of the extracellular matrix, glucose and lipid metabolism, immune evasion, invasion, and metastasis (Figure 1) [42,43]. Such biochemical alterations evidence that the hypoxic tumor microenvironment influences the majority of hallmarks of aggressive cancer behavior, translating into clinical consequences that have been associated with increased patient mortality in several cancer types [42,44].

Considering such factors, inhibiting the activity of HIF-1 and HIF-2 in hypoxic regions of cancer could potentially increase the cancer’s responsiveness to radiotherapy and/or chemotherapy [42,45], but despite the pivotal role of HIFs, only a reduced number of marine natural products have been investigated on their impact upon these heterodimeric transcription factors. Previous reviews covering natural products that impact the HIF pathway are worth mentioning, mostly covering plant-derived and synthetic inhibitors of HIF-1 with a brief mention of marine-sourced compounds [46,47,48,49,50,51,52,53]. Readers are also invited to take a glimpse at the structure–activity relationship (SAR) analysis of ten chemotypes reported to be HIF-1 inhibitors [54], as well as to the patent survey by Nakamura and colleagues, summarizing the information about patented HIF inhibitors over the time period from 2011 to 2015 [55]. 

As far as we are aware, there is only a descriptive review by Nagle and Zhou dealing with marine natural products that were identified as inhibitors of HIF-1 activation as of December 2008 [56], justifying the updated comprehensive analysis herein delivered. In the current analysis, we provide critical input as the marine natural products that are known to inhibit HIF-1 and HIF-2 are also highlighted, considering other effects that cooperate with the overall ‘anticancer potential’. Our comprehensive literature search covers the period up to February 2024 without a start date restriction, with the keywords ‘marine natural products’, ‘hypoxia-inducible factor’, and ‘cancer’, as well as cross-referencing being used to expand the search criteria.

## 2. Marine Natural Products Acting as Inhibitors of HIF-1 and HIF-2

An expanding and motivating list of hundreds of additional lead structures produced by marine organisms currently feeds the preclinical pipeline, being expected that many new agents will step into clinical trials in the upcoming years. In the following sections, a comprehensive discussion will be presented on the most promising metabolites produced by marine organisms displaying anticancer properties via impact upon the HIF pathway. 

### 2.1. Peptides

**Figure 2:**
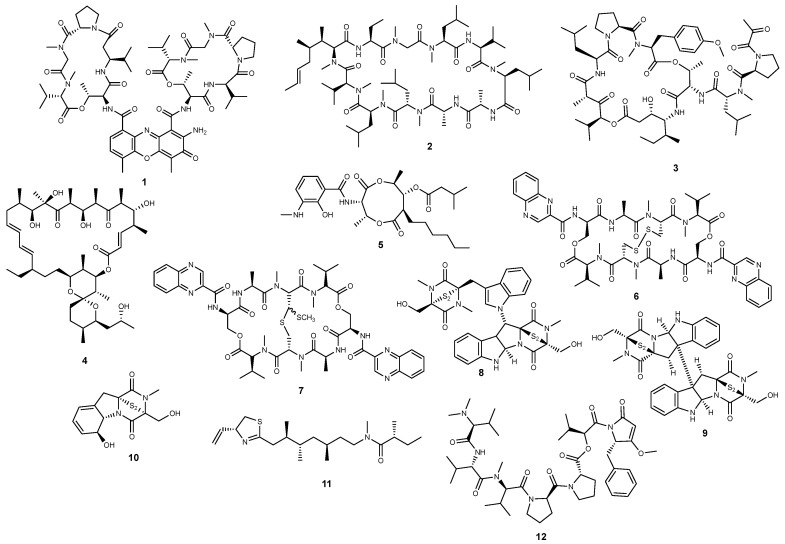
Structures of marine-derived peptides acting on the HIF pathway.

While originally discovered in terrestrial counterparts, actinomycin D (also known as dactinomycin) (**1**) (Figure 2) has also been reported from several marine strains of *Streptomycetes* [57,58]. The cyclic dipeptide antibiotic is a well-known chemotherapeutic and radiosensitizing agent (Cosmogen^®^) with anticancer effects mainly deriving from DNA intercalation and the subsequent impediment on the progression of RNA polymerases [59]. Actinomycin D (**1**) leads to an extremely fast action upon RNA polymerases but is a nonselective inhibitor of protein transcription, which determines some of the severe side effects [59]. Apart from the main anticancer mechanisms, **1** has been reported to inhibit HIF-1 binding activity to DNA in hepatocellular carcinoma Hep3B cells [60,61], but the effects in vascular smooth muscular cells demonstrated that actinomycin D (**1**) solely attenuated angiotensin II-mediated induction of HIF-1α protein expression levels and not the hypoxia-dependent induction [62].

Also, the undecacyclopeptide cyclosporin A (**2**) (Figure 2) has been reported from marine-derived isolates of fungi [63]. The blockbuster immunosuppressant stimulates the activity of peptidylprolyl hydroxylases, ultimately modifying Pro-564 of the HIF-1α protein. The outcomes include the abolishment of hypoxic stabilization of HIF-1α and HIF-1α-mediated cellular responses in glioma C6 cells, being hypothesized that cyclosporin A (**2**) might limit adaptative responses to hypoxia [64].

Unlike actinomycin D (**1**) and cyclosporin A (**2**), the second-generation didemnin plitidepsin (**3**) (Figure 2), originally isolated from the Mediterranean tunicate *Aplidium albicans*, appears to be a strictly marine-derived metabolite [65]. Plitidepsin (**3**) received market approval under the trade name Aplidin^®^ for the treatment of patients with relapsed and refractory multiple myeloma, acting as a pleiotropic chemotherapeutic agent [66]. While mainly acting as a disruptor of the translation elongation factor eEF1A2 protein complexes, leading to the induction of early oxidative stress and subsequent sustained activation of JNK in multiple myeloma cells, **3** was found to impact against angiogenic-related genes in anaplastic thyroid cancer xenografts [67]. Angiogenesis-related genes targeted by plitidepsin (**3**) include not only HIF-1 but also the transforming growth factor-*β* (TGF*β*), TGF*β*R2, melanoma growth stimulating factor 1 (GRO1), cadherin and vasostatin, cumulatively inducing tumor starvation [67].

Reported from both terrestrial and marine *Streptomyces* spp., oligomycin A (**4**) (Figure 2) is mainly reputed as a mitochondrial F0F1-ATPase inhibitor [68,69]. Microbial antibiotics have been extensively used to elucidate the mechanistic aspects of ATP formation and energy requirements in tumor cell biology [70]. The effects of oligomycin A (**4**) on short-term hypoxia were investigated on the highly resistant human uveal melanoma Mum2B and U87 glioblastoma cells, results suggesting that the anticancer effects might be enhanced by preventing HIF-1α protein accumulation [71]. 

Described as a marine natural product [72], the microbial metabolite antimycin A (**5**) (Figure 2) also acts as an inhibitor of oxidative phosphorylation, specifically through the binding to the quinone reduction site of the cytochrome *bc1* complex [73]. Relevantly, the inhibitory effects of **5** upon the mitochondrial electron transport chain were followed by the inhibition of hypoxia-dependent HIF-1α protein induction by decreasing its half-life in osteosarcoma 143B cells [74]. Inhibition of HIF-1α protein induction was further suggested to occur independently of mitochondrial reactive oxygen species (ROS) production [74]. Together with the scientific outcomes delivered by Maeda and colleagues, antimycin A (**5**) is suggested to inhibit angiogenesis through the decreased production of the vascular endothelial growth factor (VEGF) caused by inhibition of HIF-1α activation [75].

While both triostin A (**6**) and echinomycin (**7**) (Figure 2) are labeled as competent HIF-1 inhibitors, the latter has been long into the spotlight as one of the most potent HIF-1 inhibitors, as well as impacting the coactivator/DNA interaction [76,77]. The anticancer effects of **6** and **7** derive from their DNA intercalating effects through the quinoxaline chromophores, preferentially binding at *CpG* steps in the minor groove of the double helix [77]. Echinomycin (**7**) was the first bifunctional intercalating agent proceeding to clinical development, but it was discontinued due to its poor effectiveness in patients with solid tumors [78,79]. While sharing the same structural backbone, **6** and **7** differ on the intrapeptide bridge between the two cysteine residues, determining a distinct HIF-1 inhibitory capacity [80]. Echinomycin (**7**) acts as a potent inhibitor of HIF-1α, blocking HIF-1 DNA binding of endogenous nuclear proteins but mainly the binding to the canonical hypoxia-responsive element (HRE) of VEGF promoter [80,81]. Triostatin (**6**), echinomycin (**7**), and a series of synthetic derivatives were evaluated for effects on the HIF-1 transcriptional activation under hypoxic conditions and cytotoxicity on MCF-7 cells, SAR analysis indicating that the cyclic depsipeptide architecture is as an attractive scaffold to develop selective anticancer agents targeting the hypoxic tumor microenvironment [80].

While both triostin A (**6**) and echinomycin (**7**) have been mainly reported from marine strains of bacterial isolates [82], epithiodiketopiperazines are almost exclusively reported from fungi, many of which obtained from marine sources and also impacting HIF-1 signaling [83]. The diketopiperazine dimers chetomin (**8**) and chaetocin (**9**) (Figure 2) occur both as terrestrial and marine-derived metabolites, being reported from marine strains of *Chaetomium cristatum* and *Nectria inventa*, respectively [84,85]. Both **8** and **9** are recognized for their ability to target the transcriptional coactivator p300 by displacing the zinc ion from its CH1 domain (C-TAD) [86,87]. This action disrupts the interactions with the C-terminal trans-activation domain of HIF-1, ultimately resulting in the attenuation of hypoxia-inducible transcription of downstream signaling components [88,89]. While chetomin (**8**) was identified as the first naturally occurring antagonist of the C-C chemokine receptor type 2 (CCR2) [90], it has played a significant role in uncovering the mechanisms that contribute to the invasiveness of specific cancer cell types, particularly highlighting the preponderant role of hypoxia in ovarian and triple-negative breast cancers [89,91]. Furthermore, the effective inhibition of HIF-1 by chetomin (**8**) leads to a reduction in CA9 and VEGF mRNA expression, enhancing the radiation response specifically under severely hypoxic conditions in HT 1080 human fibrosarcoma and U251MG and U343MG glioma cells [88,92]. Biological implications deriving from the disruption of the HIF-1/p300 complex include a direct antitumor effect but also antiangiogenic properties, with chaetocin (**9**) being more effective than chetomin (**8**) in this matter. The epithiodiketopiperazine dimer **9** is primarily acknowledged for its function as an epigenetic agent via the pharmacological inhibition of SUV39H, being the first histone lysine methyltransferase (HKMT) inhibitor [93,94,95], but several of its anticancer effects are also attributed to the disruption of the HIF-1α/p300 complex. For instance, chaetocin (**9**) exhibited a reduction in microvessel outgrowth in the low nM range, co-immunoprecipitation experiments providing additional evidence that these effects are, at least in part, a result of inhibiting the HIF-1/p300 interaction [96]. Downstream consequences include reduced levels of secreted VEGF and subsequent downregulation of glycolytic genes, namely *LDHA* and *ENO1*, suggesting a role in inhibiting cell survival under hypoxia and promoting cell death in hypoxic areas [96].

Described as the first member of epithiodiketopiperazines, gliotoxin (**10**) (Figure 2) has been progressively reported on its anticancer ability deriving from multiple targets, including the disruption of HIF-1 activity. The structurally simple epithiodiketopiperazine is commonly sourced from terrestrial and marine-derived strains of *Aspergillus* spp. [97,98,99]. Reece et al. (2014) also described the antiangiogenic effects of **10**, indicating similar mechanisms as those observed for the dimeric epipolythiodiketopiperazines chetomin (**8**) and chaetocin (**9**): (i) disruption of the C-TAD domain of HIF-1 and (ii) downregulation of the target genes *LDHA* and *ENO1*. In contrast, gliotoxin (**10**) did not impact VEGF expression in PC3 prostate cancer cells, pointing to cell-specific effects that differ from those of **8** and **9** [96].

First described by Gerwick and colleagues as a neurotoxic agent and originally sourced from the marine cyanobacterium *Lyngbya majuscula* [100], kalkitoxin (**11**) (Figure 2) was later found to be a competent disruptor of hypoxic signaling [101]. The lipopeptide inhibited hypoxia-induced activation of HIF-1 in T47D breast ductal carcinoma cells in the low nM range, acting through the suppression of mitochondrial oxygen consumption at electron transport chain (ETC) complex I (NADH-ubiquinone oxidoreductase). Kalkitoxin (**11**) efficiently inhibited the hypoxic induction of *VEGF* or glucose transporter-1 (*GLUT-1)* mRNA expression in a concentration-dependent manner, displaying also antiangiogenic effects via the suppression of hypoxia-induced secreted VEGF protein [101].

The discovery of the pentapeptides dolastatins from the sea hare *Dolabella auricularia* prompted the development of the CD30-targeted antibody-drug conjugate brentuximab vedotin (Adcetris^®^) that is currently available for the treatment of Hodgkin lymphoma [17,102]. Dolastatin-15 (**12**) (Figure 2) was also originally reported by Pettit and colleagues from the Indian Ocean sea hare *D. auricularia* [103] but has been progressively labeled as a cyanobacterial symbiont metabolite [104,105]. The pentapeptide is predominantly reputed as a potent cytostatic agent that, along with a series of synthetic analogs, proceeded to clinical development [106,107,108,109,110,111]. Despite mainly acting as a microtubule-destabilizer [112,113], **12** also displays HIF-mediated antiangiogenic activity, with inhibitory effects upon HIF-1α being recorded in vitro and in vivo [114]. Experiments in the single knockout cells HCT116^HIF-1α−/−^ and HCT116^HIF-2α−/−^ suggested that dolastatin 15 (**12**) preferentially targeted HIF-1α but not HIF-2α, showing decrease in potency against HCT116^HIF-1α−/− HIF-2α−/−^ and HCT116^HIF-1α−/−^ in contrast to the parental and HCT116^HIF-2α−/−^ cells [114]. Luesch’s group further reported that **12** is able to suppress aberrant transcriptional upregulation of HIF-1α target genes in a zebrafish model, significantly diminishing pathological vascularization [114]. 

### 2.2. Alkaloids

**Figure 3:**
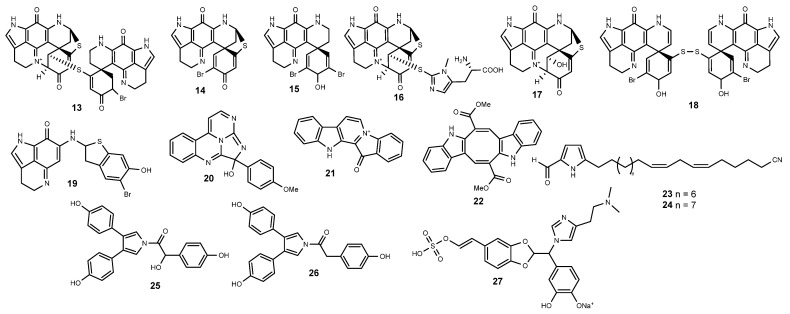
Structures of marine-derived alkaloids acting on the HIF pathway.

During a screening on the ability of more than 170 200 crude natural product extracts to inhibit the HIF-1α/p300 interaction, a series of pyrroloiminoquinone alkaloids (**13**–**19**) (Figure 3) sourced from Australian and New Zealand collections of marine sponges, *Latrunculia* sp., were identified as inhibitors of HIF-1α transcriptional activity [115]. The peculiar structural features of pyrroloiminoquinone alkaloids include the azacarbocyclic spirocyclohexanone and pyrroloiminoquinone redox active core structures that frequently underlie the reported bioactive effects [116]. Pyrroloiminoquinone alkaloids were first screened through a cell-free protein–protein assay by measuring displacement of the HIF-1α binding domain of p300 (CH1) from the p300 binding domain of HIF-1α (C-TAD), with (−)-discorhabdin B dimer (**13**), (+)-discorhabdin B (**14**), (−)-discorhabdin H (**16**), and (−)-discorhabdin L (**17**) featuring as the most effective with IC_50_ values under 5 µM [115]. Results were also obtained in three cancer cell lines transfected with an HIF-1 reporter plasmid containing a hypoxia response element that mediates HIF-1-dependent gene transcription, with all the alkaloids (**13**–**19**) proving to significantly decrease the transcriptional activity of HIF-1α in human colorectal carcinoma HCT 116 cells, and (−)-discorhabdin B dimer (**13**) featuring as the most competent [115]. While most of the sponge-derived pyrroloiminoquinone alkaloids also led to a reduction in luciferase activity in human prostate adenocarcinoma LNCaP cells, there is a clear cell-type specificity on the HIF-1α transcriptional activity, as none proved to be active in COLO 205 colon cancer cells [115]. (−)-Discorhabdin H (**16**) also interfered with the secretion of the downstream target VEGF in LNCaP cells cultured under hypoxic conditions [115]. In addition to the significant inhibition of endothelial cell proliferation and tube formation recorded in HUVEC cells, ex vivo experiments demonstrated that the antiangiogenic effects of (−)-discorhabdin L (**17**) are also related to the decrease in microvessel outgrowth, as demonstrated in the aortic ring assay, at concentrations as low as 1 µM [117].

Bioassay-guided fractionation of an extract obtained from specimens of the marine ascidian *Eudistoma* sp., collected in Palau, yielded eudistidine A (**20**) (Figure 3), bearing an uncommon heterocyclic architecture comprising two pyrimidine rings and an imidazole ring that is fused in a tetracyclic core containing guanidine, amidine, and hemiaminal functionalities [118]. Eudistidine A (**20**) blocked the binding of soluble CH1 (p300) to immobilized C-TAD (HIF-1α) with concentration dependency, with an IC_50_ value of 75 μM being estimated [118].

Fascaplysin (**21**) (Figure 3), an indole alkaloid originally discovered from the marine sponge *Fascaplysinopsis reticulata* [119], has been reported as having a pleiotropic anticancer mechanism of action, including DNA intercalation [120], inhibition of angiogenesis [121], but mainly the selective inhibition of CDK4 [121,122,123,124]. The effects on tumor angiogenesis have been further elucidated in human melanoma A375 and colorectal carcinoma HCT116 cells, as well as in an A375 cell-injected xenograft model. Both in vitro and in vivo data suggested that the antiangiogenic effects of fascaplysin (**21**) derive from a strong suppression of VEGFR2 as well as of HIF-1α and its downstream genes [125]. 

The algal pigment caulerpin (**22**) (Figure 3), first reported in the 1970s from an ether extract of a *Caulerpa* sp. [126], was later identified as an inhibitor of HIF-1α activation in a human breast tumor T47D cell-based reporter assay [127]. Caulerpin (**22**) was able to inhibit both hypoxia (1% O_2_)- and chemical hypoxia (10 μM 1,10-phenanthroline)-induced HIF-1α activation with comparable potency, and while being unable to inhibit the induction of VEGF and GLUT-1 mRNAs by 1,10-phenanthroline in human breast cancer T47D cells, there was a marked decrease on the hypoxia-derived induction of both target genes [127]. Liu et al. [127] further reported that **22** selectively suppresses mitochondrial respiration at complex I (NADH-ubiquinone oxidoreductase), suggesting that, as previously reported with other complex I inhibitors, the blockage of hypoxic induction of HIF-1α protein is mediated by the inhibition of complex III superoxide anion generation.

Chemical analysis of an extract obtained from a *Mycale* sp. sponge yielded 26 alkylpyrroles with variable HIF-1 inhibitory potency [128]. The most active lipophylic pyrroles, mycalenitrile-6 (**23**) and -7 (**24**) (Figure 3), displayed selective HIF-1 inhibitory effects using the T47D cell-based HIF-1 activation reporter assay, preferentially inhibiting hypoxia-induced HIF-1 activation in comparison to the effects on chemical hypoxia/iron chelator-induced activation [128]. Analogously to caulerpin (**22**), the inhibition of HIF-1 activation is mediated through the selective inhibition of mitochondrial respiration at complex I, and both **23** and **24** appear to prevent hypoxic mitochondria from releasing ROS signaling molecules, consequently avoiding the stabilization of HIF-1α protein [128].

Since the first report on the isolation of lamellarins from the Palauan mollusk *Lamellaria* sp. in 1985 [129], more than 50 lamellarins have been reported, mainly from *Didemnum* spp. ascidians [130,131,132,133,134,135,136,137], as well as from sponges [138,139]. Lacking the planar pentacyclic chromophore and consequently differing from the prototype structure of lamellarins, 7-hydroxyneolamellarin A (**25**) (Figure 3) was originally reported from the sponge *Dendrilla nigra* [140]. Unlike most congeners from the subfamily of neolamellarins isolated from the same source, 7-hydroxyneolamellarin A (**25**) effectively inhibited hypoxia-induced HIF-1 activation in a T47D human breast tumor cell-based reporter assay, suppressing also the activation of VEGF [140]. Effects were also demonstrated through in vivo experiments, **25** being able to suppress tumor growth of 4T1 cells in BALB/c mice by inhibiting the accumulation of HIF-1α in tumor tissue [141]. Li and colleagues further suggested that 7-hydroxyneolamellarin A (**25**) targets the protein with the ability to stabilize HIF-1α in hypoxia, as no impact on the degradation of synthesis of HIF-1α was observed [141]. While less effective than the 7-hydroxylated derivative, neolamellarin A (**26**) (Figure 3) also demonstrated HIF-1 inhibitory activity in the reporter gene assay based on T47D human breast tumor cells [140] and prompted the synthesis of a series of derivatives to identify the structural requirements underlying the effects towards HIF-1 [142]. Both naturally occurring constituents (**25** and **26**) proved to be more active than the synthetic derivatives, with SAR analysis indicating that the methoxylation of *p*-hydroxy groups diminished the HIF-1 inhibitory capacity and that a two-carbon aliphatic carbon chain linker was more favorable to the HIF-1 inhibitory activity than a single or triple carbon chain [142].

Displaying an unusual skeleton with a five-membered acetal ring, wondonin A (**27**) (Figure 3) was sourced from a two-sponge association (*Poecillastra wondoensis* and *Jaspis* sp.) collected at Keomun Island, Korea [143]. Generally, wondonins are reputed for their strong ability to suppress the expression of HIF-1α and VEGF, but what sets them apart from most other antiangiogenic agents is their remarkable ability to inhibit angiogenesis without causing significant cytotoxicity [144]. Wondonin A (**27**) reduced the expression of HIF-1a and VEGF in endothelial cells and could suppress HIF-dependent transcription in HaCaT cells. Authors suggest that the enhancement of the assembly of pVHL and HIF-1α could be the underlying mechanism causing the proteasomal degradation of HIF-1 [144]. To optimize the antiangiogenic properties of wondonins, a series of synthetic analogs have been assayed on their effects towards VEGF-induced cell growth in HUVECs, replacement of benzodioxole and imidazole moieties by benzothiazole and 1,2,3-triazole rings, respectively, resulting in enhanced effects [145].

### 2.3. Polyketides

**Figure 4:**
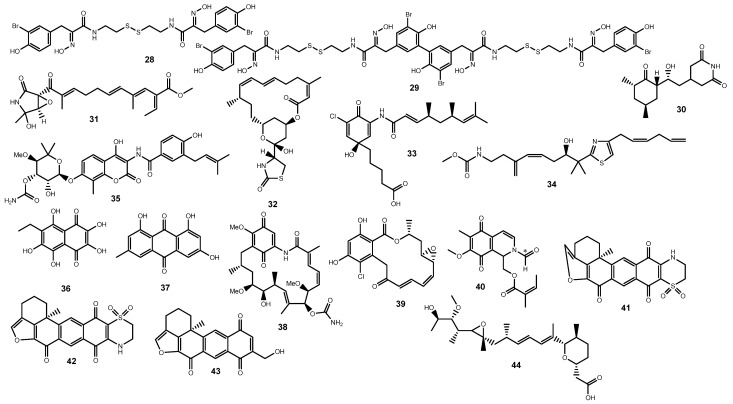
Structures of marine-derived polyketides acting on the HIF pathway.

Psammaplin A (**28**) (Figure 4) is a spongean bromotyrosine-derived dimer with intriguing anticancer properties that has been gaining increased attention since its discovery in 1987. It was originally isolated independently from three research groups, from a *Psammaplysilla sponge* [146], two unidentified sponges [147,148], and later from additional sponge species such as *Pseudoceratina purpurea* [149,150], *Aplysinella rhax* [151], and from the two-sponge association *Poecillastra wondoensis* and *Jaspis wondoensis* [152,153]. Displaying significant in vitro cytotoxicity against a wide panel of human cancer cells namely A549, SK-OV-3, SK-MEL-2, XF498, HCT15 [154], and leukemia cell lines [146,151], as well as in vivo growth inhibitory activity in an A549 lung xenograft mouse model [149], psammaplin A (**28**) has been mainly highlighted as an epigenetic modulator due to its dual inhibitory activity against the chromatin-modifying enzymes histone deacetylases (HDAC) and DNA methyltransferase (DNMT) [149]. Later, in 2015, Kim and coworkers reported psammaplin A (**28**) ability to induce autophagic cell death, markedly increasing the expression of damage-regulated autophagy modulator (DRAM), as well as causing the reduced expression of SIRT1, suggesting an association between SIRT1 expression and p53 acetylation in chemorresistant cancer cells [155]. A series of psammaplins obtained from a lipid extract sample of the sponge *Dendrilla lacunosa* were assayed on a cell-based reporter assay carried out in T47D cells transfected with pHRE-luc for HIF-1 activity. The results revealed intriguing effects with psammaplins being generally characterized by a biphasic pattern of activation, psammaplin A (**28**) strongly activating HIF-1 at 3 µM but displaying reduced effects at lower concentrations [156]. On the other hand, at concentrations ranging from 0.1 to 30 µM, the biphenylic dimer of **28**, bisaprasin (**29**) (Figure 4), inhibited HIF-1 in T47D cells [156].

Widely used in biological research, cycloheximide (**30**) (Figure 4) is mostly known as a transcription inhibitor that interferes with the translocation step and thus blocks translation elongation. Cycloheximide (**30**) was first reported as an antifungal agent by Whiffen-Barksdale of Upjohn Company in the mid-1940s from strains of *Streptomyces griseus* and remains commercially available as Actidione^®^ [157]. Marine-derived strains of Actinomycetes are also reported to display the biosynthetic machinery to produce **30** [158,159]. Cycloheximide (**30**) was first reported to potently block HIF-1α protein expression and inhibit hypoxia- and iron chelator-induced HIF-1 activation in human liver adenocarcinoma Hep3B cells [160]. Semenza et al. (1994) later demonstrated that the induction of erythropoietin, a hormone encoded by a gene whose transcription is regulated by O_2_ tension, was also attenuated in Hep3B and HeLa cells [161].

Bioassay-guided fractionation of an extract obtained from cultures of a marine isolate of *Penicillium* sp. led to the isolation of the neuritogenic compound epolactaene (**31**) (Figure 4) [162]. Epolactaene (**31**) was later found to bind human heat shock protein (HSP) 60 Cys^442^, both in in vitro and in situ settings, inhibiting its chaperone activity [163] and supporting the identification of HSP60 as a regulator of the hypoxia-inducible factor subunit HIF-1α [163,164].

First described 50 years ago, the 16-membered macrolide toxin latrunculin A (**32**) (Figure 4) was early reported as a microfilament disruptor [165]. Originally isolated from the sponge *Latruncula magnifica* [166,167], the toxin was also reported from *Spongia mycofijiensis* [168] and curiously from associated nudibranchs [168,169]. Latrunculin A (**32**) forms reversible complexes with G-actin, preventing its polymerization, ultimately leading to the disruption of microfilament organization, with no effect on the organization of the microtubular system [165,170]. A potent anti-invasive activity was also observed in prostate cancer PC-3M cells treated with **32**, attenuating their invasiveness and cell migration and selectively suppressing hypoxia-induced HIF-1 activation in T47D breast tumor cells [171]. Relevantly, the blockage of actin dynamics by latrunculin A (**32**), through the inhibition of either polymerization or depolymerization, appears to underlie the attenuation of HIF-1α protein expression via the mTOR/p70^S6K^/Mdm2 signaling pathway in a p53-independent manner [171,172]. 

Chemical analyses of an extract and fractions obtained from a saltern-derived halophilic *Streptomyces* strain collected on Shinui Island in the Republic of Korea yielded a series of cytotoxic salternamides [173,174]. Reported as the first chlorinated compound in the manumycin family, salternamide A (**33**) (Figure 4) displayed strong cytotoxicity towards a panel of cancer cell lines, being particularly active towards human colorectal carcinoma HCT-116 cells and human gastric carcinoma SNU638 cells, with IC_50_ values below 1 μM [173]. The anticancer activity of **33** towards HCT116 cells appears to derive from two distinct mechanisms involving G2/M cell cycle arrest and subsequent apoptotic cell death, but also via suppression of HIF-1α translation through the modulation of the mTOR signaling and the downregulation of the axis of the PI3K/ STAT3 signaling pathways [175].

Originally reported by Crews’s group in the sponge *Spongia mycofijiensis* [176] and later from the sponge *Dactylospongia* sp. [177], the thiazolo-polyene mycothiazole (**34**) (Figure 4) was initially described as an anthelminthic agent. NCI 60 tumor cell panel screening revealed a potent cytotoxic effect against several cancer cell lines with selective nM potency toward lung cancer cells, namely SCLC and NSCLC cancer lines [178]. Analogously to other HIF-1 inhibitors, **34** selectively disrupts the mitochondrial electron transport chain by suppressing mitochondrial respiration at complex I (NADH-ubiquinone oxidoreductase) and potently blocking hypoxia-induced HIF-1 activation (IC_50_ = 1 nM) [179]. Additionally, mycothiazole (**34**) was found not only to cause a decrease in ROS levels but also to suppress the hypoxic induction of the HIF-1 target genes, VEGF and GLUT-1 [179]. While its nM cytostatic effect in sensitive cells showing a hypoxic response is explained through the disruption of mitochondrial function and inhibition of mitochondrial electron transport complex I, a biphasic response was observed in some sensitive cells, suggesting an additional target. Furthermore, the mycothiazole (**34**) effect in mitochondrial genome-knock out ρo insensitive HL-60, LN18, and Jurkat cells, not affected by mitochondrial electron transport chain suppression, leads to a cytotoxic effect rather than cytostatic at µM concentrations, suggesting the involvement of a distinct nonmitochondrial mechanism of action [178].

Previously used in clinical settings as an antistaphylococcal agent [180], the clinical utility of novobiocin (**35**) (Figure 4) continues to be repurposed, particularly as a chemotherapeutic agent [181,182]. Novobiocin (**35**) was first obtained from cultures of the actinomycete *Streptomyces niveus* [183,184,185] but is also known to occur in marine counterparts [186]. The antibiotic has been in the spotlight as a first-in-class polymerase theta (Polθ) inhibitor, acting as a noncompetitive inhibitor of ATP hydrolysis, and it is currently under clinical development in patients with tumors that harbor aberrant DNA repair genes [182,187]. However, the anticancer mechanism of **35** has long been recognized as being pleiotropic and also includes the inhibition of HSP90 autophosphorylation by interacting with a C-terminal ATP-binding pocket, consequently hampering hypoxia-induced HIF-1α accumulation [188,189,190]. Antiproliferative effects of novobiocin (**35**) in A549 and MCF-7 cells appear to derive from the disruptions between the HIF1α CTAD and p300 CH1 complex, which downregulates the transcriptional activation of HIF-responsive genes such as CA9, being also able to downregulate the mRNA expression of Akt1 and mTOR in both cell lines [191].

Currently available in Russia, echinochrome A (**36**) (Figure 4) is used as an active substance of the drug Histochrome^®^, clinically used on the prophylaxis of reperfusion damages after myocardial infarction for treating ischemia and infarction in acute forms and also as retinoprotector for dystrophic damages of the retina and diabetic retinopathy, for cataract, keratitis, and uveitis [192,193,194,195]. The polyhydroxylated 1,4-naphthoquinone was first described as a bactericidal agent by Mac Munn in 1885 from the coelomocytes of the sea urchin *Echinus esculentus* [196,197] but was recently reported to act as a SOD3 mimetic, controlling the expression of cell enzymes through the interference with HIFs, but also enhancing the transcription of PPAR-α and the coactivator1 PPAR-γ (PCG-1 α) [198].

While emodin (**37**) (Figure 4) is mainly reported from several plant genera [199,200], it is also commonly documented as a fungal natural product [201]. The anthraquinone has been isolated from several marine-derived strains of fungi [202,203], with Gomes and colleagues reporting its isolation from cultures of a marine-sponge-associated strain of *Eurotium cristatum* [204]. Emodin (**37**) is widely reputed for its anticancer effects and the interference with a series of molecular events underlying cancer development and progression [205]. The interference with the HIF-1 pathway emerges as a common mechanism underlying an array of potential therapeutic effects of **37**, ranging from neuroprotection and anti-inflammatory effects to the preservation of intestinal barrier function and amelioration of pulmonary inflammation [206,207,208,209,210]. Similarly, the response to the hypoxic environment in the cancer microenvironment has also been commonly reported. Emodin (**37**) inhibited HIF-1α expression in five human pancreatic cancer cell lines, as well as attenuating cancer cachexia in in vivo models. Treatment of athymic mice xenografted with MiaPaCa2 cells resulted in diminishing cancer cell growth and enhancing energy homeostasis through the improvement of cancer-induced hepatic gluconeogenesis and skeletal muscle wasting [211]. Emodin (**37**) also demonstrated in vitro and in vivo suppressive effects against anaplastic thyroid cancer by affecting TRAF6-mediated pathways. Besides blocking angiogenesis by inhibiting the TRAF6/HIF-1α/VEGF pathway in 8505c and SW1736 cells, **37** also suppressed anaplastic thyroid cancer metastasis by inhibiting the TRAF6/CD147/MMP9 pathway [212]. Relevant effects were also noted in hypoxia-induced radioresistance in HepG2 cells, with emodin (**37**) synergistically improving irradiation effects through the inhibition of hypoxia-induced signaling factors such as HIF-1α and histone demethylase (JMJD1A), but mainly via increased PARP1 cleavage, activation of caspase-9, and inhibition of JMJD2B [213].

Labeled as the first naturally occurring HSP90 inhibitor and originally reported in the culture filtrates of *Streptomyces hygroscopicus* var. *geldanus* var. *nova* [214], the ansamycin antibiotic geldanamycin (**38**) (Figure 4) is commonly described in marine strains of *Streptomyces* [215,216,217,218]. The pivotal work of Whitesell and Neckers demonstrated that **38** inhibited the formation of a *v*-Src–HSP90 complex through binding to the ATP-binding site in the N-terminal domain of HSP90 [219,220]. Geldanamycin (**38**), the inaugural HSP90 inhibitor to undergo clinical trials, was unable to progress further clinical development due to its marked hepatotoxicity, likely linked to the electrophilic methoxybenzoquinone group [221]. As observed in prostate cancer PC-3 and LNCaP cells, the interaction of **38** with the N-terminal ATP binding domain of HSP90 induces destabilization and degradation of numerous HSP90 client proteins by the ubiquitin–proteasome pathway, among them HIF-1α [222,223]. Both in vitro studies and xenograft animal models employing various human tumor cell lines have demonstrated the therapeutic promise of geldanamycin (**38**) in cancer treatment, particularly in solid cancer types, not only due to potent cytotoxicity but also due to a significant decrease in cell invasion deriving from HIF-1α-mediated effects [224,225,226,227].

Despite bearing a dissimilar structural architecture, radicicol (**39**) (Figure 4) shares a close mechanistic similarity with geldanamycin (**38**), disrupting the folding of protein kinases dependent on HSP90, and implying the degradation of the client protein HIF-1α [220]. While less commonly reported from marine sources, Crews’ group described the isolation of **39** from an EtOAc extract obtained from cultures of the fungus *Humicola fuscoatra* isolated from sediments collected in Tutuila, American Samoa [228].

Convergent with the late identification of HIF-2 as a potential target for the development of alternative chemotherapeutic agents, only a limited number of marine-derived candidates have been identified as inhibitors. McKee and collaborators screened over 146,000 extracts of plants, microorganisms, and marine invertebrates on their effects upon HIF-2, and only three extracts from soft corals scored positively [229], ten sponge extracts being also selected for further studies [230]. The inseparable mixture of isomers *N*-formyl-1,2-dihydrorenierone (**40a/40b**) (Figure 4), isolated from *Haliclona velinea*, displayed selectivity for the HIF-2α induced transcription of mRNAs [230].

The potential of adociaquinones A (**41**) and B (**42**) (Figure 4) as lead structures for the development of chemotherapeutic agents dates back to 1988 with their discovery from an unspecified sponge from the genus *Adocia* and the first report on their in vitro cytotoxic effect against cancer cell lines [231]. Later, and being isolated from a *Xestospongia* sp. MeOH extract, both xestoquinones were found to inhibit topoisomerase II [232]. In a recent report, the bioassay-guided isolation of an extract of the sponge *Petrosia alfiani* yielded a new structurally related quinone, 14-hydroxymethylxestoquinone (**43**) (Figure 4), along with **41** and **42** [233]. Adociaquinones A (**41**) and B (**42**) selectively suppressed iron chelator-induced HIF-1 activation in T47D cells, each with IC_50_ values of 0.2 μM, and led to the suppression of secreted VEGF protein by 1,10-phenanthroline, causing a moderate increase in secreted VEGF under hypoxic condition. While both **41** and **42** promoted oxygen consumption without affecting mitochondrial membrane potential, 14-hydroxymethylxestoquinone (**43**) acted as a protonophoric uncoupler of oxidative phosphorylation and decreased mitochondrial membrane potential [233]. Furthermore, while the majority of the Cdc25B inhibitors are quinones, adociaquinone B (**42**) attracted special and increased attention due to its potent and remarkable selectivity, being identified as the most potent known Cdc25B inhibitor [234].

Herboxidiene (**44**) (Figure 4) was originally described as a metabolite of *Streptomyces chromofuscus* by researchers in Monsanto Company [235], being later rediscovered in cultures of the *Streptomyces* sp. GMY01 strain isolated from a marine sediment sample collected from Krakal beach in Yogyakarta, Indonesia [236]. On the initial reports, herboxidiene (**44**) displayed cytotoxicity in the low nM range against several human tumor cell lines, with the main mode of action being attributed to the targeted effects upon SF3B and interference with spliceosome assembly [237,238]. Cooperating with the effects towards SF3B1, **44** also causes a dual impact of the signaling mediated by VEGFR2 and the expression of HIF-1α, inhibiting the transcription and splicing of *HIF-1α* mRNA in HUVECs [239]. Furthermore, the antiangiogenic effects of herboxidiene (**44**) are also suggested based on the inhibition of neovascularization of the chorioallantoic membrane in developing chick embryos [239].

### 2.4. Phenolics

**Figure 5:**
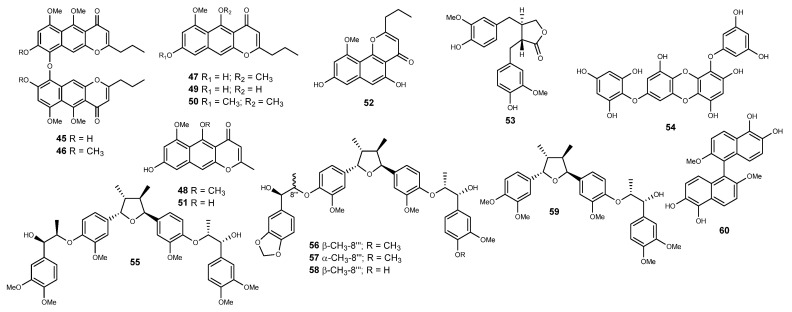
Structures of marine-derived phenolics acting on the HIF pathway.

Bioassay-guided fractionation of a CH_2_Cl_2_/MeOH extract obtained from samples of the marine crinoid *Comantheria rotula* afforded a series of benzochromenones that were assayed on their inhibitory effects upon HIF-1α in cell-based reporter assay [240]. The benzochromenones **45**–**52** (Figure 5) significantly inhibited HIF-1 activation in T47D breast tumor cells with IC_50_ values ranging from 1.7 to 7.3 μM and from 0.6 to 3.0 μM for hypoxia-induced and 1,10-phenanthroline-induced activation, respectively [240]. However, solely comaparvin (**52**) led to a decrease in the hypoxic induction of secreted VEGF proteins. When assayed on the NCI 60-cell line panel, the dimeric neocomantherin derivative **45** decreased tumor cell growth with a low level of selectivity, but TMC-256A1 (**51**) was characterized by a unique pattern of anticancer activity as indicated by the COMPARE analysis [240].

While matairesinol (**53**) (Figure 5) has been restrictively described as a plant lignan, Urbatzka’s group reported the dibenzylbutyrolactone lignan in hexane extracts obtained from the leaves and stems of the marine seagrass *Halophila stipulacea* [241]. Now also labeled as a marine natural product, **53** reduced hypoxia-induced accumulation of HIF-1α protein with concentration dependency, with no effects upon the synthesis of cytoskeletal (tubulin) or cell cycle (cyclin D1) proteins in HeLa cells, inhibiting also tumor-conditioned media-induced angiogenesis via the diminished expression of VEGF [242]. Matairesinol (**53**) demonstrated efficient suppression of hypoxia and VEGF-induced angiogenesis at concentrations lower than those required to hinder HUVEC growth, suggesting that it might selectively disrupt angiogenic signaling pathways by suppressing mitochondrial ROS generation [242].

The phlorotannin 7-phloroeckol (**54**) (Figure 5) is a common metabolite of *Ecklonia* kelps and has been progressively reported as being active towards a series of biologically relevant targets [243,244,245,246]. 7-Phloroeckol (**54**) is acknowledged to inhibit tumor angiogenesis in HepG2 cells and HUVECs via inhibition of HIF-1α protein expression and the secretion of VEGF protein by blocking PI3K/AKT/mTOR/P70S6K and RAS/MEK/ERK/MNK mediated signal transduction pathways [247]. 

Chemical analysis of the aquatic plant *Saururus cernuus* led to the isolation of a series of neolignans with nM potency towards HIF-1α [248,249]. Manassantins A (**55**), B (**56**), and B_1_ (**57**) (Figure 5) potently inhibited hypoxia-activated HIF-1 with IC_50_ values of 3 nM, while 4-*O*-demethylmanassantin B (**58**) and 4-*O*-methylsaucerneol (**59**) (Figure 5) were weaker on their inhibitory capacity (IC_50_ values of 30 and 20 nM, respectively) [248,249]. The SAR analysis by Nagle and colleagues evidenced that the absence of both hydroxylated side chain segments is an essential structural requirement for the HIF-1 inhibitory activity by this series of lignans, absence of one side chain phenylpropyl unit, as in the sesquilignan **59**, reducing also the inhibitory potency [249]. Additionally, the replacement of the methylenedioxyl moiety by *O*-methyl groups and the difference in relative configuration of at least one of the two side chains in **56** and **57** have only a slight influence on the potency of HIF-1 inhibition [249]. Hypoxic induction of VEGF was attenuated by the manassantins **55**–**58**, with manassantin A (**55**) and B_1_ (**57**) also blocking the hypoxia-induced increase in *CDKN1A* and *GLUT-1* mRNA levels [249].

In an attempt to elucidate the constituents underlying the effects of an extract obtained from a *Lendenfeldia* sp. sponge on hypoxia-induced HIF-1 activation in T47D breast tumor cells, Dai and colleagues carried out the isolation of a series of structurally dissimilar constituents [250]. In addition to a series of active constituents, the naphthalene dimer **60** (Figure 5) also significantly inhibited both hypoxia- and 1,10-phenanthrolin-induced HIF-1 activation in T47D breast tumor cells, with an IC_50_ value of 4.3 µM [250].

### 2.5. Terpenes

**Figure 6:**
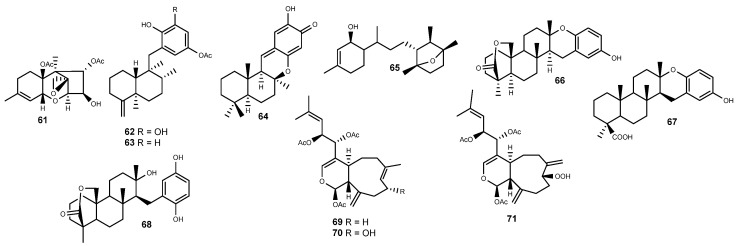
Structures of marine-derived sesquiterpenes (**61**–**64**) and diterpenes (**65**–**71**) acting on the HIF pathway.

The trichothecene-type mycotoxin diacetoxyscirpenol (**61**) (Figure 6) is a well-known metabolite from phytopathogenic *Fusarium* spp. [251,252]. However, it was also reported from a marine bacterial parasite *Bacillus licheniformis* isolated from the red alga *Gelidium pacificum* [253]. While several mycotoxins are able to upregulate the HIF pathway [254], diacetoxyscirpenol (**61**) dampened the activation of hypoxic genes by HIF, thereby reducing anchorage-independent colony formation, endothelial tube formation, and tumor growth in mice [253]. The impact of **61** upon HIF-1 is suggested to occur either through the inhibition of HIF-1α translation or its dimerization with ARNT, as well as by hampering the hypoxia-induced production of VEGF [253].

Kobayashi’s group reported the isolation of several sesquiterpene phenols, namely dictyoceratins A (also known as smenospondiol) (**62**) and C (**63**) (Figure 6) from the sponge *Dactylospongia elegans* [255]. While **62** was originally described from an Okinawan *Hippospongia* sp. [256], its congener dictyoceratin C (**63**) was initially reported from a *Dactylospongia* sp. [257], both being reported later from the sponges *Polyfibrospongia australis* [258] and *Spongia* sp. [259]. Both **62** and **63** were originally described as antimicrobial agents; however, they were found later to display hypoxia-selective antiproliferative effects, inhibiting the proliferation of human prostate cancer DU145 cells under hypoxic conditions in low µM concentrations [255] and also showing a strong antitumor effect in mice xenografted with sarcoma S180 cells upon oral administration [260,261]. Molecular studies performed with dictyoceratins A (**62**) and C (**63**) revealed that their selective antiproliferative activity was derived from their inhibitory effects toward HIF-1α, inhibiting the accumulation of HIF-1α in hypoxia-adapted DU145 cells [255]. Kawachi and coauthors further detailed the mode of action of dictyoceratins A (**62**) and C (**63**), demonstrating that both HIF inhibitors bind to RNA polymerase II-associated protein 3 (RPAP3) in the vicinity of the TRP1 domain and disturbed the R2TP/PEDL/HSP90 complex, consequently leading to dysfunction of mTOR and the reduced accumulation of HIF-1 [262].

The bioassay-guided screening on HIF-2 inhibitors by McKee and coworkers also led to the isolation of the meroterpene puupehenone (**64**) (Figure 6) from the sponge *Hyrtios reticulatus* [230]. However, analogously to the remaining HIF-2 inhibitors isolated from the active sponge extracts, puupehenone’s (**64**) effect on VEGF secretion was coupled with a decrease in total protein, suggesting that it was related to cellular toxicity [230]. The sesquiterpene quinone and several analogs have been repeatedly described in various sponges from distinct genera such as *Heteronema* [263], *Hyrtios* [264,265,266,267], *Stronglyphora* [268], *Xestospongia* [269], *Dysidea* [270,271] and from *Dactylospongia* [272]. Concomitantly, **64** has also been documented to have a wide range of biological properties, namely as a potent and selective human 5-lipoxygenase inhibitor [267,273]. Puupehenone (**64**) was further assayed as a potential antiangiogenic agent, inhibiting the endothelial cell differentiation in bovine aortic endothelial (BAE) cells in vitro with an IC_50_ value of 10 ± 2 μM, but without apparent selectivity since, at the same range of concentrations, it was also cytotoxic against a panel of human cancer cell lines [274]. Interestingly, puupehenone (**64**) was also reported as useful in tumor immunotherapy, being attached to a modified antigenic peptide derived from Melan-A/MART-1 protein, frequently recognized by MHC class I-restricted CD8+ cytotoxic T-lymphocytes (CTL). Despite the low affinity for HLA-A2 molecules, the resulting adduct fitted on the TCR/HLA-A2 interface, leading to the stimulation of CTL [275].

Displaying an uncommon 7-oxabicyclo[2.2.1]heptane ring system, lauranditerpenol (**65**) (Figure 6) was reported from an extract of samples of the red alga *Laurencia intricata* collected in Discovery Bay, Jamaica [276]. The algal metabolite enhanced the degradation of HIF-1α in breast cancer T47D cells, with an IC_50_ value as low as 0.4 μM, exhibiting selective anticancer effects under hypoxic conditions without affecting normoxic cell growth [276]. Lauranditerpenol (**65**) also inhibited the mitochondrial electron transport pathway as a complex I inhibitor, evidencing that the inhibitory effects on hypoxia-induced HIF-1 activation were dependent on the increase in cellular O_2_ availability under hypoxia [276].

The chemical family strongylophorines has been gaining attention since the first report on the isolation of the first members from the sponge *Strongylophora durissima* in the late 1970s [277]. Several strongylophorines have been into the spotlight due to the reports on their versatile modes of action towards cancer cells, acting as Rho-dependent inhibitors of tumor cell invasion [278], proteasome inhibitors [279], and as inhibitors of the HIF-1 transcriptional pathway [280]. The bioassay-oriented isolation of strongylophorine-2, -3, and -8 (**66**–**68**) (Figure 6) from the MeOH extract obtained from the sponge *Petrosia (Strongylophora) strongylata* enabled their identification as inhibitors of the HIF-1-dependent luciferase expression in U251-HRE glioblastoma cells without interference with luciferase expression in U251-pGL3 control cells [280]. While the study was limited to strongylophorine-2, -3, and -8 (**66**–**68**) and chromanol, a preliminary SAR analysis suggests that the presence of the A-ring lactone is a structural requirement that enhances the HIF-1 inhibitory activity of strongylophorines [280]. Besides their HIF-1 selectivity, the strongylophorines **66**–**68** also effectively decreased the expression of VEGF [280]. 

Chemical analysis of an extract obtained from the soft coral *Asteropicularia laurae*, collected from a large reef west of Mabul in Malaysia, yielded a series of cembrane diterpenes [229]. While most proved to be inactive, 13-*epi*-9-deacetylxenicin (**69**), 13-*epi*-9-deacetoxyxenicin (**70**) and its hydroperoxide (**71**) (Figure 6) effectively suppressed HIF-2α activity in renal carcinoma 786-0 cells with IC_50_ values of 6.2, 3.4, and 11.8 μM, without exhibiting significant cytotoxicity [229].

**Figure 7:**
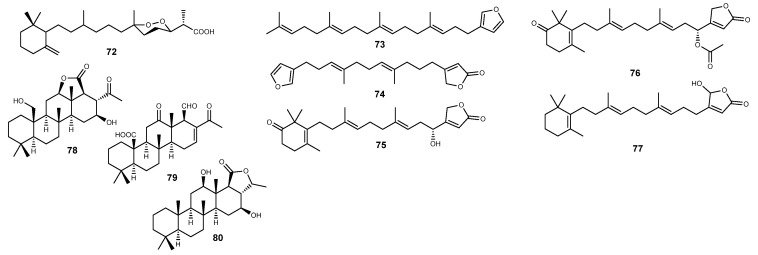
Structures of marine-derived sesterterpenes acting on the HIF pathway.

Diacarnoxide B (**72**) (Figure 7), a norsesterterpene peroxide isolated from the Papua New Guinea sponge *Diacarnus levii*, was found to inhibit hypoxia-induced HIF-1 activation in T47D cells with an IC_50_ value of 12.7 μM [281]. Interestingly, while diacarnoxide B (**72**) inhibited prostate DU145 and PC-3 cells and breast MCF-7 and MDA-MB-231 cell proliferation, both under normoxic and hypoxic conditions, it caused an unusual enhanced and selective inhibitory effect at low concentration in MCF-7 and MDA-MB-231 cells under hypoxic conditions [281].

In addition to its potent and selective inhibitory effect toward Cdc25A [282] and in vitro cytotoxicity against a set of isogenic HCT-116 colon cancer cell lines [283], the spongean metabolite furospinosulin-1 (**73**) (Figure 7) has been claiming much attention for its hypoxia-selective growth inhibitory effect. Furospinosulin-1 (**73**) is a furanosesterterpene originally described from an *Ircinia* sp. sponge more than 50 years ago [284] but has also been widely reported from other spongean genera such as *Hippospongia* [256], *Fasciospongia* [285], *Spongia* [282,286], and *Smenospongia* [283,287]. In 2010, on the report on its isolation from the Indonesian sponge *Dactylospongia elegans*, **73** was found to lead to concentration-dependent and selective suppression of human prostate cancer DU145 cells under hypoxic conditions, as well as displaying in vivo antitumor effects in mice xenografted with mouse sarcoma S180 cells, with no side effects being recorded upon oral administration [288]. Curiously, despite its hypoxia-selective growth inhibitory effect, furospinosulin-1 (**73**) did not inhibit the accumulation of HIF-1 or VEGF, instead modulating the activation of several genes involved in the hypoxia signaling pathway. The selective growth inhibitory effect of **73** against hypoxia-adaptable cancer cells was initially attributed to the suppression of the insulin-like growth factor-2 (IGF-2) gene transcription, selectively induced under hypoxic conditions [288]. Recently, the same group further detailed the mechanism of action of furospinosulin-1 (**73**), reporting an effective and selective effect against hypoxic regions of tumors, stemming from the direct binding to the transcriptional regulators p54^nrb^ and LEDGF/p75, both known as mediators of hypoxia adaptation in cancer cells [289].

Discovered more than 35 years ago as a metabolic product of the Red Sea sponge *Dysidea herbacea* [290] and later from the sponges *Lendenfeldia* sp. [291] and *Spongia officinalis* [292], furospongolide (**74**) (Figure 7) was reported as the first marine-derived furanolipid able to inhibit hypoxia-induced HIF-1 activation [291]. During a screening on extracts from natural sources with the ability to inhibit HIF-1 activation, the lipid extract of *Lendenfeldia* sp. was found to be active, and the subsequent bioassay-guided isolation afforded three scalarene-type sesterterpenes with similar activity and selectivity profile as HIF-1 inhibitors along with furospongolide (**74**) [291]. Unlike the co-isolated sesterterpenes displaying significant cytotoxicity, only **74** preferentially inhibited HIF-1 activation with a 10-fold selectivity compared to its antiproliferative/cytotoxic effect against T47D cells, as well as blocking VEGF induction. The HIF-1 inhibitory activity of furospongolide (**74**) is mediated through the suppression of tumor cell respiration via the blockade of NADH-ubiquinone oxidoreductase (complex I)-mediated mitochondrial electron transfer, consequently blocking the HIF-1 transcription regulator protein HIF-1α [291].

Bioassay-guided isolation of the Palau sponge *Hyrtios communis* afforded several sesterterpenes with HIF-1 inhibitory activity [293]. Among them, the new sesterterpenes thorectidaeolide A (**75**), 4-acetoxythorectidaeolide A (**76**), and the previously reported luffariellolide (**77**) (Figure 7) were found to be the most potent inhibitors of hypoxia-induced HIF-1 activation with IC_50_ values of 3.2, 3.5, and 3.6 μM, respectively [293]. Unlike the other sesterterpenes, **77** displayed a significant antiproliferative effect in T47D and MDA-MR-231 breast cancer cells [293]. Luffariellolide (**77**) has been previously reported in several sponge species from the genera *Luffariella* [294], *Cacospongia* [295], *Acanthodendrilla* [296], and *Thorectandra* [297], being initially reported as a reversible phospholipase A2 inhibitor [294] and later as a weak inhibitor of the protein tyrosine phosphatase Cdc25 [298]. More relevant, its in vitro cytotoxicity against human cancer cell lines was attributed to its agonistic effect on retinoic acid receptors (RARs) [299]. Unlike other RAR ligands, luffariellolide (**77**) adopts a distinct binding mode in RARα through a covalent modification with its unusual i-hydroxybutenolide ring terminus, stabilizing the interaction of RARs with its ligands. It displayed significant cytotoxic effects against monocytic leukemia and promyeloid leukemic cell lines, as well as against MCF-7 breast cancer cells [299]. Furthermore, **77** was also effective against the retinoic acid-resistant colon cancer HCT-116 cell line, inducing the expression of the tumor suppressors RARβ and CRABPII [299].

In addition to the naphthalene dimer **60** (Figure 5), the homoscalarane sesterterpenes **78**–**80** (Figure 7) were obtained from the lipid extract of Indonesian samples of *Lendenfeldia* sp. [250]. While the three homoscalarane sesterterpenes proved to be efficient inhibitors of hypoxia-induced HIF-1 activation in T47D cells, **79** featured as the most active, with an IC_50_ value as low as 0.64 µM [250], evidencing that the free C-25 aldehyde moiety is essential for HIF-1 inhibition and that its lactonization markedly decreases the inhibitory potency [250].

**Figure 8:**
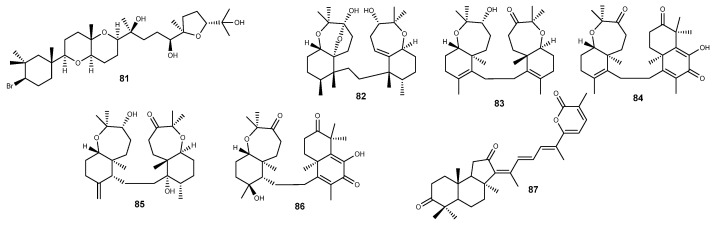
Structures of marine-derived triterpenoids acting on the HIF pathway.

Mainly reported from the red algae *Laurencia* spp. [300,301,302,303,304,305], thyrsiferol (**81**) (Figure 8) selectively suppresses mitochondrial respiration at complex I, inhibiting also the hypoxia-induced HIF-1 activation in T47D human breast tumor cells at the same concentration (3 µM) [306]. Roussis and colleagues further described the ability to counteract the hypoxic induction of the HIF-1 target genes *VEGF* and *GLUT-1* in a concentration-dependent manner [306]. The variance in cytotoxicity observed between the sensitive breast cancer T47D cells, which heavily depend on mitochondrial oxidative phosphorylation, and the relatively unresponsive breast cancer MDA-MB-231 cells, which primarily utilize glycolysis, can be explained by the impact of thyrsiferol (**81**) on mitochondrial function [306].

A series of cytotoxic triterpenoids bearing an uncommon condensed oxepane-cyclohexane scaffold have been widely reported in sponges from the genera *Axinella* [307,308,309,310,311,312,313,314] and *Ptilocaulis* [311,315], with several members from this series of spongean terpenoids being reported as HIF-1 inhibitors. Sodwanone V (**82**) (Figure 8) is featured as the most active, inhibiting both hypoxia- and phenanthroline-induced HIF-1 activation in T47D breast tumor cells and being the only sodwanone derivative suppressing hypoxia-induced HIF-1 activation in PC-3 prostate tumor cells [314]. Displaying weaker inhibitory effects, with IC_50_ values in the range 20–25 µM, sodwanone T (**83**) and 10,11-dihydrosodwanone B (**84**) (Figure 8) inhibited both hypoxia- and phenanthroline-induced HIF-1 activation in T47D cells, while 3-*epi*-sodwanone K (**85**) and sodwanone A (**86**) (Figure 8) could only inhibit the hypoxia-induced activation [314].

There is an increasing number of reports describing the anticancer effects of stellettin B (**87**) (Figure 8), an isomalabaricane-type triterpene commonly reported from sponges of the genera *Geodia* [316,317], *Jaspis* [318,319], *Rhabdastrella* [320,321], and *Stelletta* [322,323]. Stellettin B (**87**) appears to act mainly due to the induction of autophagy and apoptosis by interfering with the PI3K/Akt, Stat3, and mTOR signaling pathways in glioblastoma [324,325] and chronic myeloid leukemia cells [319]. The antiproliferative effects upon the oral squamous cell carcinoma cells OC2 and SCC4 derive from autophagic cell death, dropping the expression levels of p62 and increasing Beclin-1 and LC3-II levels [326]. In addition to the modes of action mentioned above, **87** treatment led to antiangiogenic effects in human glioblastoma U87MG and GBM8401 cells, being observed to significantly downregulate p-Stat3 and HIF-1α and culminating in the diminished expression and secretion of VEGF [325]. The antiangiogenic effects of stellettin B (**87**) were confirmed in two in vivo models, with VEGF mRNA expression being decreased in zebrafish embryos and reduction in angiogenesis also being recorded in murine Matrigel Plug models [325].

## 3. Discussion

As demonstrated above, a considerable number of marine natural products prove to be effective in interfering with HIFs and might have potential utility in treating various types of tumors (Table S1), particularly those with a negative prognosis due to hypoxia-induced resistance and metastasis. However, as already demonstrated, some are accompanied by severe toxicity that limits their translation to clinical use, while others are in the preliminary phase of characterizing their mechanisms of action and validation in disease models more indicative of therapeutic utility.

For example, the clinical development of diacetoxyscirpenol (**61**) ceased after a phase II clinical trial for cancer treatment demonstrated severe side effects [327,328]. However, it should be noted that **61** proved to inhibit HIF-1 at concentrations 20-fold lower than those displaying cytotoxicity [253,329]. The clinical development of echinomycin (**7**) was also discontinued due to the lack of anticancer efficacy in patients with solid tumors [330,331,332], but its incorporation in liposomes has been recently suggested as a safe and effective therapeutic option, profiting from the HIF-1α inhibition in metastatic cancers [333]. The same applies to gliotoxin (**10**), whose clinical use has been precluded despite its discovery more than 80 years ago. Its clinical use has been revisited in recent years, with a particular focus on investigating the possible strategies to reduce toxicity, such as the targeted delivery of **10** through nanoparticles [334] or the use of lower doses in combination with other anticancer drugs [335]. In other instances, as in the case of kalkitoxin (**11**), it is possible to anticipate toxicity even in the absence of clinical data, as the sodium channel and mitochondria-associated neurotoxicity may limit the therapeutic potential [101]. It is also unlikely that radicicol (**39**) might become a chemotherapeutic agent despite the HSP90 inhibitory ability and anticancer effects observed in vitro, as it was found to be devoid of any in vivo activity in animal models [336].

Certainly, there will be other limitations, particularly those inherent in obtaining active constituents from marine natural sources that are scarce or produced in limited quantities. The inhibitory mode of action of strongylophorine-2 (**66**) for the HIF-1 oriented transcription pathway has not been properly elucidated because of the scarcity of isolating the same from natural sources, prompting its total synthesis [337]. However, there will still be cases where the synthesis of synthetic analogs may not overcome limitations inherent to the structural architecture itself. For example, wondonin (**27**) and derivatives are highly unstable in acidic environments due to the five-membered acetal ring and the vinyl sulfate moieties [145].

However, in analogy to the numerous successful cases associated with the development of drugs inspired by natural molecules, particularly those obtained from marine sources, many of the HIF inhibitors presented here are *bona fide* leads for the translation into therapy. This could occur either in their original structural form or, more likely, through the synthesis of synthetic derivatives optimized for efficacy, toxicity, and pharmacokinetic parameters.

## Data Availability

Not applicable.

## Supplementary Materials

The following supporting information can be downloaded at: https://www.mdpi.com/article/10.3390/md22040143/s1, Table S1: Marine derived HIF inhibitors.
