# Supplementary material for: Marine-Derived Leads as Anticancer Candidates by Disrupting Hypoxic Signaling through Hypoxia-Inducible Factors Inhibition
Source: Mar Drugs. 2024 Mar 23;22(4):143. doi: 10.3390/md22040143 (PMC11051506; doi:10.3390/md22040143)
Supplement: Supplementary file 1 [file marinedrugs-22-00143-s001.zip › marinedrugs-2926788-supplementary.pdf]

Table S1. Marine natural products impacting HIF signalling pathways

Number	Compound	Source of isolation	References
**Peptides**			
**1**	Actinomycin D	Bacteria	[57, 58]
**2**	Cyclosporin A	Fungi	[63]
**3**	Aplidin	Tunicate	[65]
**4**	Oligomycin A	Bacteria	[68, 69]
**5**	Antimycin A	Bacteria	[72]
**6**	Triostin A	Bacteria	[82]
**7**	Echinomycin	Bacteria	[82]
**8**	Chetomin	Fungi	[84, 85]
**9**	Chaetocin	Fungi	[84, 85]
**10**	Gliotoxin	Fungi	[97-99]
**11**	Kalkitoxin	Cyanobacteria	[100]
**12**	Dolastatin-15	Sea hare	[103]
**Alkaloids**			
**13**	Discorhabdin B dimer	Sponge	[115]
**14**	Discorhabdin B	Sponge	[115]
**15**	3-Dihydrodiscorhabdin C	Sponge	[115]
**16**	Discorhabdin H	Sponge	[115]
**17**	Discorhabdin L	Sponge	[115]
**18**	Discorhabdin W	Sponge	[115]
**19**	Makaluvamine F	Sponge	[115]
**20**	Eudistidine A	Ascidian	[118]
**21**	Fascaplysin	Sponge	[119]
**22**	Caulerpin	Algae	[126]
**23**	Mycalenitrile-6	Sponge	[128]
**24**	Mycalenitrile-7	Sponge	[128]
**25**	7-Hydroxyneolamellarin A	Sponge	[140]
**26**	Neolamellarin A	Sponge	[142]
**27**	Wondonin	Sponge	[143]
**Polyketides**			
**28**	Psammaplin A	Sponge	[146-153, 156]
**29**	Bisaprasin	Sponge	[156]
**30**	Cycloheximide	Bacteria	[157]
**31**	Epolactaene	Fungi	[162]
**32**	Latrunculin A	Sponge; Nudibranch	[166-169]
**33**	Salternamide A	Bacteria	[173, 174]
**34**	Mycothiazole	Sponge	[176, 177]
**35**	Novobiocin	Bacteria	[183-186]
**36**	Echinochrome A	Sea urchin	[196, 197]
**37**	Emodin	Fungi	[201-204]
**38**	Geldamycin	Bacteria	[214-218]
**39**	Radicicol	Fungi	[228]
**40**	*N*-formyl-1,2-dihydrorenierone	Sponge	[230]
**41**	Adociaquinone A	Sponge	[231-233]
**42**	Adociaquinone B	Sponge	[231-233]

**43**	14-Hydroxymethylxestoquinone	Sponge	[233]
**44**	Herboxidiene	Bacteria	[235, 236]
**Phenolics**			
**45**	Coumarin dimer-1	Crinoid	[240]
**46**	Coumarin dimer-2	Crinoid	[240]
**47**	Coumarin-1	Crinoid	[240]
**48**	Coumarin-2	Crinoid	[240]
**49**	Coumarin-3	Crinoid	[240]
**50**	Coumarin-4	Crinoid	[240]
**51**	TMC-256A1	Crinoid	[240]
**52**	Comaparvin	Crinoid	[240]
**53**	Matairesinol	Marine seagrass	[241]
**54**	7-Phloroeckol	Algae	[243-246]
**55**	Manassantin A	Aquatic plant	[248, 249]
**56**	Manassantin B	Aquatic plant	[248, 249]
**57**	Manassantin B_1	Aquatic plant	[248, 249]
**58**	4-*O*-Demethylmanassantin B	Aquatic plant	[248, 249]
**59**	4-*O*-methylsaucerneol	Aquatic plant	[248, 249]
**60**	Naphthalene dimer	Sponge	[250]
**Sesquiterpenes**			
**61**	Diacetoxyscirpenol	Fungi; Bacteria	[251-253]
**62**	Dictyoceratin A	Sponge	[255-259]
**63**	Dictyoceratin C	Sponge	[255-259
**64**	Puupehenone	Sponge	[230, 263-272]
**Diterpenes**			
**65**	Laurenditerpenol	Algae	[276]
**66**	Strongylophorine-2	Sponge	[280]
**67**	Strongylophorine-3	Sponge	[280]
**68**	Strongylophorine-8	Sponge	[280]
**69**	13-*epi*-9-Deacetoxyxenicin	Coral	[229]
**70**	13-*epi*-9-Deacetylxenicin	Coral	[229]
**71**	13-*epi*-9-Deacetoxyxenicin peroxide	Coral	[229]
**Norsesterterpenes**			
**72**	Diacarnoxide B	Sponge	[281]
**Furanosesterterpenes**			
**73**	Furospinosulin-1	Sponge	[256, 282-288]
**74**	Furospongolide	Sponge	[290-292]
**Sesterterpenes**			
**75**	Thorectidaeolide A	Sponge	[293]
**76**	Acetoxythorectidaeolide A	Sponge	[293]
**77**	Luffariellolide	Sponge	[293-297]
**78**	Homoscalarane sesterterpene-1	Sponge	[250]
**79**	Homoscalarane sesterterpene-2	Sponge	[250]
**80**	Homoscalarane sesterterpene-3	Sponge	[250]

Triterpenoids

**81**	Thyrsiferol	Algae	[300-305]
**82**	Sodwanone V	Sponge	[307-315]
**83**	Sodwanone T	Sponge	[307-315]
**84**	10,11-Dihydrosodwanone B	Sponge	[307-315]
**85**	3-epi-Sodwanone K	Sponge	[307-315]
**86**	Sodwanone A	Sponge	[307-315]
**87**	Stellettin B	Sponge	[316-323]
